# Supplementary material for: Empirical methods for controlling false positives and estimating confidence in ChIP-Seq peaks
Source: BMC Bioinformatics. 2008 Dec 5;9:523. doi: 10.1186/1471-2105-9-523 (PMC2628906; doi:10.1186/1471-2105-9-523)
Supplement: Additional File 1 — A variety of html documents from the USeq web site detailing the available applications, their best usage, output file type descriptions, command line menus, etc. [file 1471-2105-9-523-S1.zip › USeqUserGuides/JavaDoc.zip!/JavaDoc/util/bio/annotation/class-use/Coordinate.html]

Uses of Class util.bio.annotation.Coordinate


|  |  |  |
| --- | --- | --- |
| **Overview** \| **Package** \| **Class** \| **Use** \| **Tree** \| **Deprecated** \| **Index** \| **Help** | |  |
| PREV   NEXT | **FRAMES**    **NO FRAMES**     **All Classes** |  |


---


## **Uses of Class util.bio.annotation.Coordinate**

| Packages that use Coordinate | |
| --- | --- |
| **expr** | Classes and tools related to expression array analysis. |
| **trans.anno** | For associating intervals, binding regions, and binding peaks with GFF annotation. |
| **util.bio.annotation** | For representing gene annotation. |
| **util.bio.parsers** | Parsers and extractors, fasta, quality, text, gff.... |

Uses of Coordinate in expr

| Methods in expr that return Coordinate | |
| --- | --- |
| `Coordinate` | `ExpressedGene.getCoordinates()` |

Constructors in expr with parameters of type Coordinate

`ExpressedGene(java.lang.String name,
Coordinate coordinates,
float[] values)`

Uses of Coordinate in trans.anno

| Methods in trans.anno with parameters of type Coordinate | |
| --- | --- |
| `void` | `SubtractRegions.mask(Coordinate[] coor, java.io.File toSave)` |

Uses of Coordinate in util.bio.annotation

| Subclasses of Coordinate in util.bio.annotation | |
| --- | --- |
| `class` | `ScoredCoordinate` |

| Methods in util.bio.annotation that return Coordinate | |
| --- | --- |
| `static Coordinate[]` | `Coordinate.parseFile(java.io.File picksFile, int subStart, int subEnd)`             Parses a tab delimited chr,start,stop file. |
| `static Coordinate[]` | `Coordinate.readBinary(java.io.File file)`             Reads a binary Coordinate[] file. |
| `static Coordinate[]` | `Coordinate.subtract(Coordinate forReduction, Coordinate mask)`             Clips the forReduction based on the mask. |

| Methods in util.bio.annotation with parameters of type Coordinate | |
| --- | --- |
| `int` | `Coordinate.bpsIntersection(Coordinate other)`             Returns -1 for no overlap, 0 for complete overlap, or a positive int for the # bases of overlap. |
| `static int` | `Coordinate.findLastBase(Coordinate[] chromSpecific)`             Finds the last base. |
| `boolean` | `Coordinate.intersects(Coordinate other)`             Assumes coordinates are inclusive. |
| `static ScoredCoordinate[]` | `ScoredCoordinate.makeRanked(Coordinate[] c)`             Returns an array of ScoredCoordinate where the score is the index position of the Coordinate[]. |
| `static java.util.HashMap<java.lang.String,Coordinate[]>` | `Coordinate.splitByChromosome(Coordinate[] sortedCoordinates)`             Split by chromosome into a HashMap of chromosome:Coordinate[]. |
| `static Coordinate[]` | `Coordinate.subtract(Coordinate forReduction, Coordinate mask)`             Clips the forReduction based on the mask. |
| `static boolean` | `Coordinate.writeBinary(Coordinate[] c, java.io.File file)`             Writes a binary Coordinate[]. |

Uses of Coordinate in util.bio.parsers

Constructors in util.bio.parsers with parameters of type Coordinate

`UCSCGeneLine(java.lang.String name,
Coordinate corr)`

---


|  |  |  |
| --- | --- | --- |
| **Overview** \| **Package** \| **Class** \| **Use** \| **Tree** \| **Deprecated** \| **Index** \| **Help** | |  |
| PREV   NEXT | **FRAMES**    **NO FRAMES**     **All Classes** |  |


---
